# Metabolites of Ginger Component [6]-Shogaol Remain Bioactive in Cancer Cells and Have Low Toxicity in Normal Cells: Chemical Synthesis and Biological Evaluation

**DOI:** 10.1371/journal.pone.0054677

**Published:** 2013-01-30

**Authors:** Yingdong Zhu, Renaud F. Warin, Dominique N. Soroka, Huadong Chen, Shengmin Sang

**Affiliations:** Center for Excellence in Post-Harvest Technologies, North Carolina Agricultural and Technical State University, North Carolina Research Campus, Kannapolis, North Carolina, United States of America; National Cancer Institute at Frederick, United States of America

## Abstract

Our previous study found that [6]-shogaol, a major bioactive component in ginger, is extensively metabolized in cancer cells and in mice. It is unclear whether these metabolites retain bioactivity. The aim of the current study is to synthesize the major metabolites of [6]-shogaol and evaluate their inhibition of growth and induction of apoptosis in human cancer cells. Twelve metabolites of [6]-shogaol (M1, M2, and M4–M13) were successfully synthesized using simple and easily accessible chemical methods. Growth inhibition assays showed that most metabolites of [6]-shogaol had measurable activities against human cancer cells HCT-116 and H-1299. In particular, metabolite M2 greatly retained the biological activities of [6]-shogaol, with an IC_50_ of 24.43 µM in HCT-116 human colon cancer cells and an IC_50_ of 25.82 µM in H-1299 human lung cancer cells. Also exhibiting a relatively high potency was thiol-conjugate M13, with IC_50_ values of 45.47 and 47.77 µM toward HCT-116 and H-1299 cells, respectively. The toxicity evaluation of the synthetic metabolites (M1, M2, and M4–M13) against human normal fibroblast colon cells CCD-18Co and human normal lung cells IMR-90 demonstrated a detoxifying metabolic biotransformation of [6]-shogaol. The most active metabolite M2 had almost no toxicity to CCD-18Co and IMR-90 normal cells with IC_50_s of 99.18 and 98.30 µM, respectively. TUNEL (Terminal deoxynucleotidyl transferase dUTP nick end labeling) assay indicated that apoptosis was triggered by metabolites M2, M13, and its two diastereomers M13-1 and M13-2. There was no significant difference between the apoptotic effect of [6]-shogaol and the effect of M2 and M13 after 6 hour treatment.

## Introduction

Despite enormous efforts made toward the development of cancer therapies over the past several decades, cancer is still a major public health problem worldwide. Increasing evidence has shown that treatments using specific agents or inhibitors that target only one biological event or a single pathway usually fail in cancer therapy [1]. Conventional chemotherapeutic agents have been shown to be associated with unacceptable toxicity. New approaches to the control of cancer are critically needed. Chemoprevention is an innovative area of cancer research that focuses on the prevention of cancer through pharmacologic, biologic, and nutritional interventions [2]. Accumulating studies have shown that dietary phytochemicals present in plants and fruits, which are generally considered as non-toxic agents, can activate or block multiple important pathways that are implicated in cancer cell survival and growth [1], [3], [4]. Chemoprevention by edible phytochemicals is now considered to be a safe, inexpensive, readily acceptable and accessible approach to cancer prevention, control and management.

**Figure 1:**
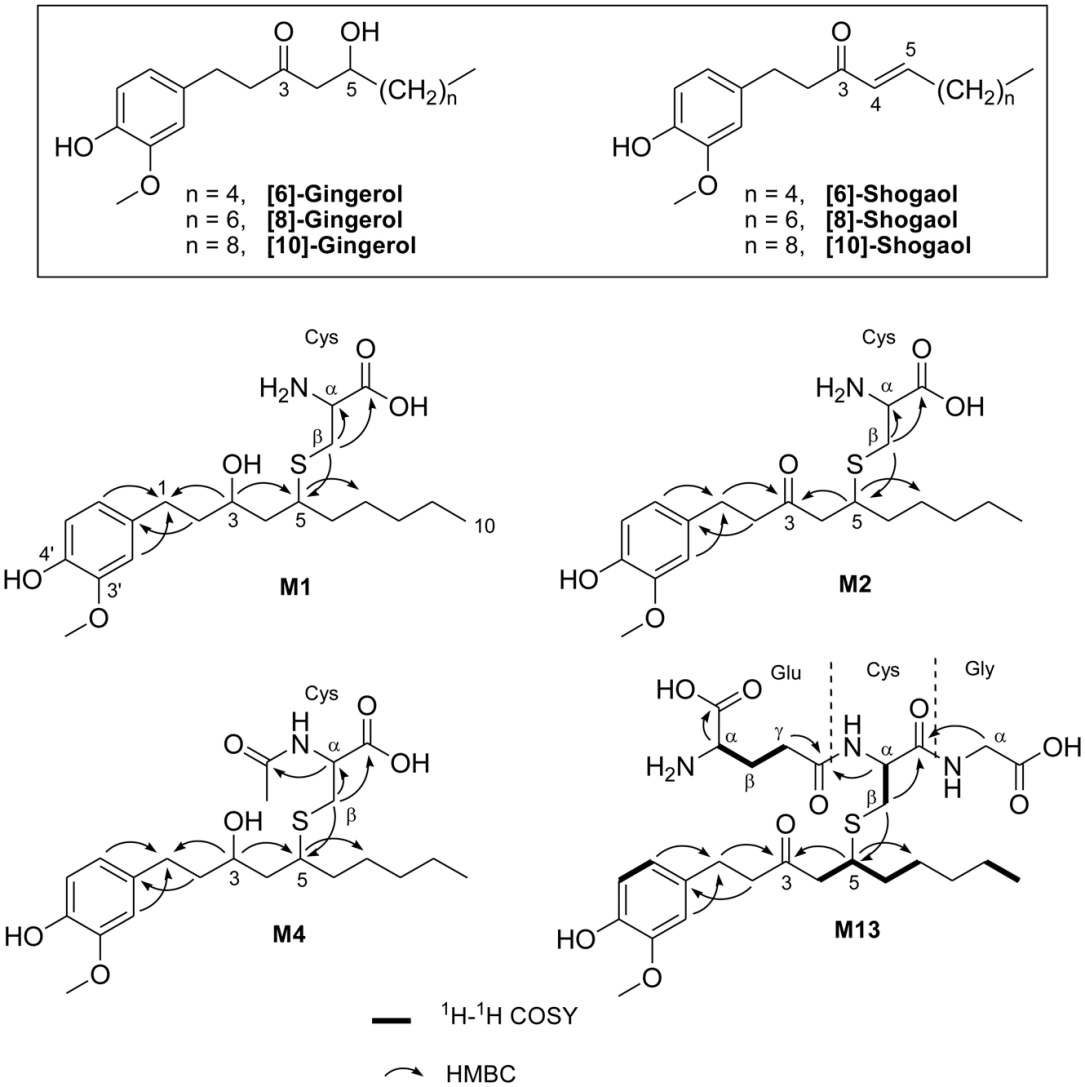
Structures of the major gingerols and shogaols in ginger; and key HMBCcorrelations of thiol-conjugates M1, M2, and M4; and key ^1^H-^1^H COSY and HMBC correlations of M13.

Ginger, the rhizome of *Zingiber officinale*, has been a commonly used spice and crude drug for many years throughout the world. Ginger is touted for its relief of nausea, anti-inflammatory properties, and putative anti-cancer activity [5]–[7]. The major pharmacologically active components of ginger are gingerols and shogaols (Figure 1) [5]. Thermal processing of gingerols gives shogoals as the primary constituents of dried ginger [5], which frequently show greater anti-carcinogenic activity than their precursors. For example, our group demonstrated that [6]-, [8]-, and [10]-shogaols exhibited much higher anti-proliferative potency than [6]-, [8]-, and [10]-gingerols against H-1299 human lung and HCT-116 human colon cancer cells [8]. Kim *et al*. reported that [6]-shogaol exhibited much stronger growth inhibitory effects in A-549 human lung cancer cells, HCT-15 human colon cancer cells, SK-OV-3 human ovarian cancer cells, and SKMEL-2 human skin cancer cells than [4]-, [6]-, [8]-, and [10]-gingerols [9]. Along with our collaborators, we have found that [6]-shogaol was more effective than [6]-gingerol in inhibiting 12-*O*-tetradecanoylphorbol 13-acetate (TPA)-induced tumor promotion in mice [10]. A more recent study found that [6]-shogaol induced apoptosis, caspase activation, and Poly (ADP-ribose) polymerase (PARP) cleavage in both SMMC-7721 human hepatocellular carcinoma cells and SMMC-7721 tumor xenografts through an endoplasmic reticulum (ER) stress-associated mechanism [11].

A recent study from our group has demonstrated that [6]-shogaol is extensively metabolized in mice and in cancer cells, in which thirteen metabolites (M1–M13) were detected and identified [12]. Trace amount of metabolites M6–M12 were purified from fecal samples collected from [6]-shogaol treated mice, and their structures were characterized based on the analysis of ^1^H, ^13^C, and 2-D NMR data [12]. Structures of the remaining metabolites (M1–M5 and M13) were elucidated based on the analysis of their MS^n^ (n = 1−3) spectra and comparison to authentic standards. With its α,β-unsaturated ketone structure, we found that [6]-shogaol can be reduced to generate metabolites M6–M9 and M11. We also found that [6]-shogaol is the substrate for thiol conjugation through the mercapturic acid pathway to form metabolites M1−M5, M10, M12, and M13. Since most of [6]-shogaol is metabolized *in vivo* and in cancer cells, the critical question is whether the metabolites of [6]-shogaol are bioactive. Even if less potent than [6]-shogaol, they may still contribute to the overall biological activity of [6]-shogaol *in vivo*.

The current challenge to study the bioactivity of metabolites is their lack of commercial availability. With trace amounts of metabolites purified from mouse fecal samples, we were only able to investigate the effects of the two major metabolites M9 and M11 on the growth inhibition and induction of apoptosis in human cancer cells. Our results showed that M9 and M11 are bioactive compounds that can inhibit cancer cell growth and induce apoptosis in human lung and colon cancer cells, albeit with less potency than [6]-shogaol. Therefore, it is timely to develop chemical methods to synthesize the metabolites of [6]-shogaol and to further examine their anti-cancer activities. The current study describes a chemical synthesis of the previously identified metabolites of [6]-shogaol and a bioactivity analysis in cancer cells. Also investigated are the toxicities of [6]-shogaol and its metabolites in normal human lung and colon cells, providing a comparison of [6]-shogaol’s toxicity in a non-cancer model.

## Results

### Chemical Synthesis of the Metabolites of [6]-shogaol

**Figure 2:**
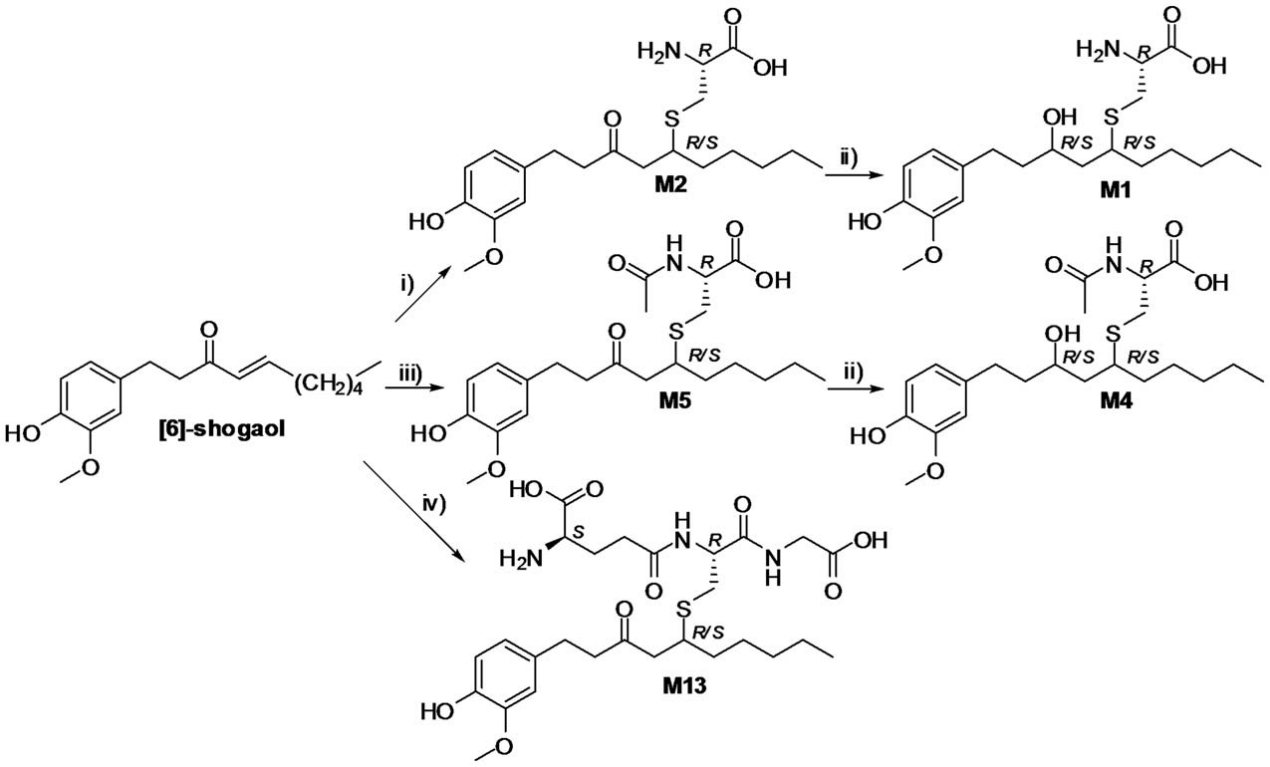
Synthesis of thiol-conjugates M1, M2, M4, M5, and M13. Reagents and conditions: i) L-cysteine, NaHCO3 (cat.), MeOH/H2O, rt, 24 h; ii) NaBH4, MeOH, 0°C, 2 h; iii) N-acetyl-L-cysteine, NaHCO3 (cat.), MeOH/H2O, rt, 72 h; iv) L-glutathione reduced, NaHCO3 (cat.), MeOH/H2O, rt, 3 h.

**Figure 3:**
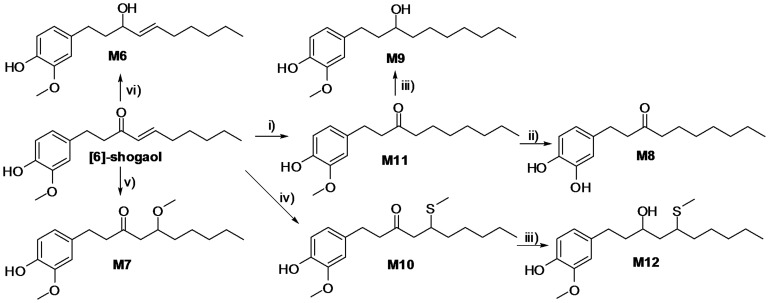
Synthesis of metabolites M6–M12. Reagents and conditions: i) H2, Pd/C (10% w/w), THF, rt, 18 h; ii) BBr3, DCM, −78°C -rt, 2 h; iii) NaBH4, MeOH, 0°C -rt, 2 h; iv) aq. 15% NaSCH3, THF, rt, 6 h; v) Na, MeOH, 0°C -rt, 4 h; vi) NaBH4, CeCl3.7H2O, MeOH, −78°C, 30 min.

Twelve metabolites (M1, M2, and M4–M13) were synthesized successfully from [6]-shogaol using simple and easily accessible semisynthetic approaches in the current study (Figures 2 and 3). In brief, reaction of [6]-shogaol with L-cysteine (Cys), N-acetyl-L-cysteine (NAC) or L-glutathione (GSH), generated thiol-conjugates M2, M5, or M13, respectively (Figure 2). Subsequently, reduction of thiol-conjugates M2 or M5 by NaBH_4_ led to hydroxylated conjugates M1 or M4, respectively (Figure 2). Selective reduction of [6]-shogaol by a combination of NaBH_4_ and CeCl_3_.7H_2_O resulted in M6 (Figure 3) [13]. Hydrogenation of [6]-shogaol on Pd/C gave M11, followed by treatment with NaBH_4_ to produce M9 (Figure 3). In addition, demethylation of M11 using BBr_3_ gave M8 (Figure 3). Michael reaction of [6]-shogaol with NaOMe or NaSMe produced the methoxy adduct M7 or the methylthio adduct M10, respectively (Figure 3) [14], [15]. Of which, the methylthio adduct M10 was treated with NaBH_4_ to give M12 (Figure 3). Since both the reduction of ketone and the Michael reactions used in this study are non-stereoselective, metabolites M1, M2, M4–M7, M9, M10, M12, and M13 are synthesized as mixtures of diastereomers.

We have fully characterized the structures of M5–M12 using their 1-D and 2-D NMR and mass spectral data in our previous study [12]. Therefore, the structures of these synthetic compounds were confirmed by comparison of their ^1^H and ^13^C NMR spectra with those of authentic standards obtained from mouse fecal samples. Structures of the remaining synthetic metabolites (M1, M2, M4, and M13), previously deduced by multi-stage mass spectrometry techniques [12], were further confirmed by their 1-D and 2-D NMR spectra data for the first time.

M2 showed the molecular formula C_20_H_31_NO_5_S on the basis of positive APCI-MS at *m/z* 398 [M+H]^+^ and its ^1^H and ^13^C NMR data. The molecular weight of M2 was 42 mass units less than that of N-acetylcysteine conjugated [6]-shogaol (M5) [12] indicating M2 was the cysteine conjugated [6]-shogaol. This was in agreement with the fact that M2 was made by [6]-shogaol and L-cysteine. This was also supported by the observation of the absence of an acetyl group in the ^1^H and ^13^C NMR spectra of M2. The linkage of an L-cysteinyl moiety to the [6]-shogaol residue at C-5 was established by HMBC cross-peaks between H_Cys_-β (δ_H_ 3.18 and 2.84) and C-5 (δ_C_ 42.3) (Figure 1). Therefore, M2 was confirmed to be 5-cysteinyl-[6]-shogaol.

M1 had the molecular formula of C_20_H_33_NO_5_S on the basis of positive APCI-MS at *m/z* 400 [M+H]^+^ and its ^1^H and ^13^C NMR data. The molecular weight of M1 was 2 mass units higher than that of M2, matching with the fact that M1 was a ketone-reduced product of M2, and also supported by the appearance of oxygenated methines (two sets of protons for the diastereomers at δ_H_ 3.66 and δ_H_ 3.90; and δ_C_ 69.3) in its ^1^H and ^13^C NMR spectra. Key HMBC correlations between H-3 (δ_H_ 3.66 and δ_H_ 3.90) to C-1 (δ_C_ 32.5) and C-5 (δ_C_ 43.8), as well as H-1 (δ_H_ 2.68 and 2.58) to C-3 (δ_C_ 69.3) in M1 (Figure 1), established a hydroxyl group at C-3 on the alkyl side chain of M1. HMBC cross-peaks between H_Cys_-β (δ_H_ 3.15 and 2.85) to C-5 (δ_C_ 43.8), and H-5 (δ_H_ 2.94) to C_Cys_-β (δ_C_ 32.8) provided the linkage of the cysteinyl moiety and C-5 position of M1 through a thioether bond. Thus, M1 was confirmed to be 5-cysteinyl-M6.

M4 showed the molecular formula C_22_H_35_NO_6_S on the basis of positive APCI-MS at *m/z* 442 [M+H]^+^ and its ^1^H and ^13^C NMR data. The molecular weight of M4 was 2 mass units higher than that of M5 (5-*N*-acetylcysteinyl-[6]-shogaol), complying with the fact that M4 was a ketone-reduced product of M5. This was further supported by the appearance of oxygenated methines (two sets of protons for the diastereomers at δ_H_ 3.70 and δ_H_ 3.88; and δ_C_ 69.3) in its ^1^H and ^13^C NMR spectra, the disappearance of the expected ketone carbonyl group in [6]-shogaol, and the key HMBC correlations observed at H-3 (δ_H_ 3.70 and δ_H_ 3.88) to C-1 (δ_C_ 32.5) and C-5 (δ_C_ 43.8), as well as H-1 (δ_H_ 2.67 and 2.58) to C-3 (δ_C_ 69.3) in its HMBC spectrum (Figure 1). The acetyl group was shown attached to α-NH_2_ of the cysteinyl moiety by HMBC correlation detected at H_Cys_-α (δ_H_ 4.54) to CH_3_ CO (δ_C_ 174.0). In addition, HMBC cross-peaks between H_Cys_-β (δ_H_ 3.00 and 2.80) and C-5 (δ_C_ 43.8) provided the linkage of an acetylcysteinyl moiety and the C-5 position of M4. M4, thereof, was confirmed to be 5-*N*-acetylcysteinyl-M6.

M13, having the molecular formula C_27_H_41_N_3_O_9_S on the basis of positive APCI-MS at *m/z* 584 [M+H]^+^ and its NMR data, was made by [6]-shogaol and reduced L-glutathione (GSH). ^1^H-^1^H COSY cross-peaks found at H_Glu_-α/H_Glu_-β/H_Glu_-γ, in combination with key HMBC correlations between H_Glu_-α (δ_H_ 3.65) to Glu α-COOH (δ_C_ 174.0) as well as H_Glu_-γ (δ_H_ 2.55 and 2.51) to Glu γ-CON (δ_C_ 175.2), recognized the structure of a glutamyl residue (Glu) (Figure 1). The structure of the cysteinyl residue (Cys) was established by ^1^H-^1^H COSY cross-peaks at H_Cys_-α/H_Cys_-β in combination with HMBC correlation between H_Cys_-β (two sets of protons at δ_H_ 3.05–2.95 and 2.84–2.80) to Cys α-CON (δ_C_ 175.2) (Figure 1). Subsequently, the connection of the glutamyl residue with the cysteinyl moiety was established between Glu γ-COOH and Cys α-NH_2_ through an amide bond, by HMBC correlations found at H_Cys_-α (δ_H_ 4.50) to Glu γ-CON (δ_C_ 175.2). The attachment of a glycinyl moiety to the cysteinyl residue was found between Cys α-COOH and Gly α-NH_2_, by HMBC correlations observed at H_Gly_-α (δ_H_ 3.80) to Cys α-CON (δ_C_ 175.2). Thus, the GSH residue was undoubtedly identified as γ-glutamyl-cysteinylglycine. Consequently, linkage of the GSH moiety to the [6]-shogaol residue was established at C-5 through a thioether bond by HMBC correlations found at H_Cys_-β (two sets of protons at δ_H_ 3.05–2.95 and 2.84–2.80) to C-5 (δ_C_ 42.2). Therefore, M13 was confirmed to be 5-glutathiol-[6]-shogaol.

**Figure 4:**
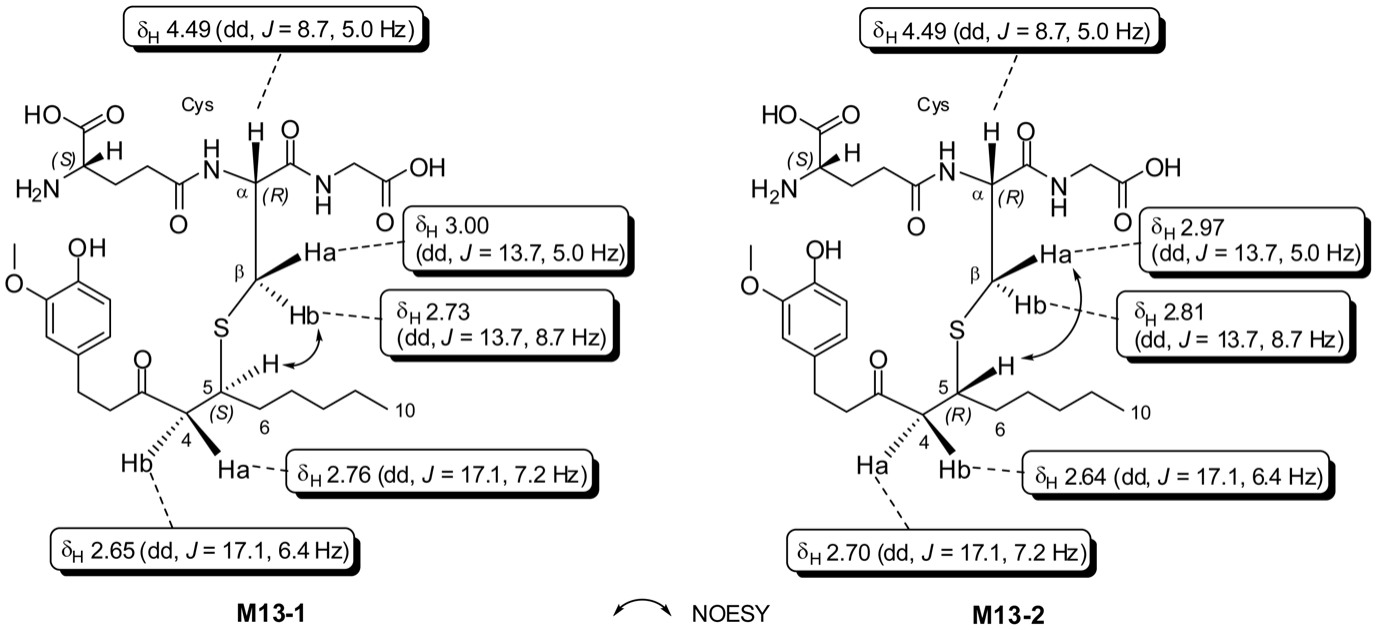
Key NOESY correlations in M13-1 and M13-2 and the major differences of the ^1^H and ^13^C NMR data of M13-1 and M13-2.

Separation of M13 isomers on preparative HPLC resulted in two diastereoisomers, M13-1 and M13-2, which had very similar ^1^H and ^13^C NMR spectra. The major differences were the ^1^H signals for H_Cys_-βb (δ_H_ 2.73 in M13-1 vs. 2.81 in M13-2) and H-4a (δ_H_ 2.76 in M13-1 vs. 2.70 in M13-2) and the ^13^C signals for C-4 (δ_C_ 49.9 in M13-1 vs. 49.6 in M13-2) and C-5 (δ_C_ 42.0 in M13-1 vs. 42.4 in M13-2) (Figure 4). In their NOESY spectra, we observed the correlations between H_Cys_-βb (δ_H_ 2.73) and H-5 (δ_H_ 3.10) in M13-1 and H_Cys_-βa (δ_H_ 2.97) and H-5 (δ_H_ 3.10) in M13-2, suggesting that H-5 in M13-1 had the same configuration as that of H_Cys_-βb and H-5 in M13-2 had the same configuration as that of H_Cys_-βa (Figure 4). It is known that H_Cys_-α in GSH residue has the *R* configuration (Figure 4). The coupling constant (*J* = 5.0 Hz) of H_Cys_-βa (δ_H_ 2.97) with H_Cys_-α (δ_H_ 4.49) is much smaller than that (*J* = 8.7 Hz) of H_Cys_-βb (δ_H_ 2.81) with H_Cys_-α, suggesting that H_Cys_-βa (δ_H_ 2.97) has the same *R* configuration as that of H_Cys_-α and H_Cys_-βb has the *S* configuration. Therefore, the configurations of H-5 in M13-1 and M13-2 were tentatively assigned as *S* and *R*, respectively (Figure 4).

### Growth Inhibitory Effects against Human Cancer and Normal Cells

**Figure 5:**
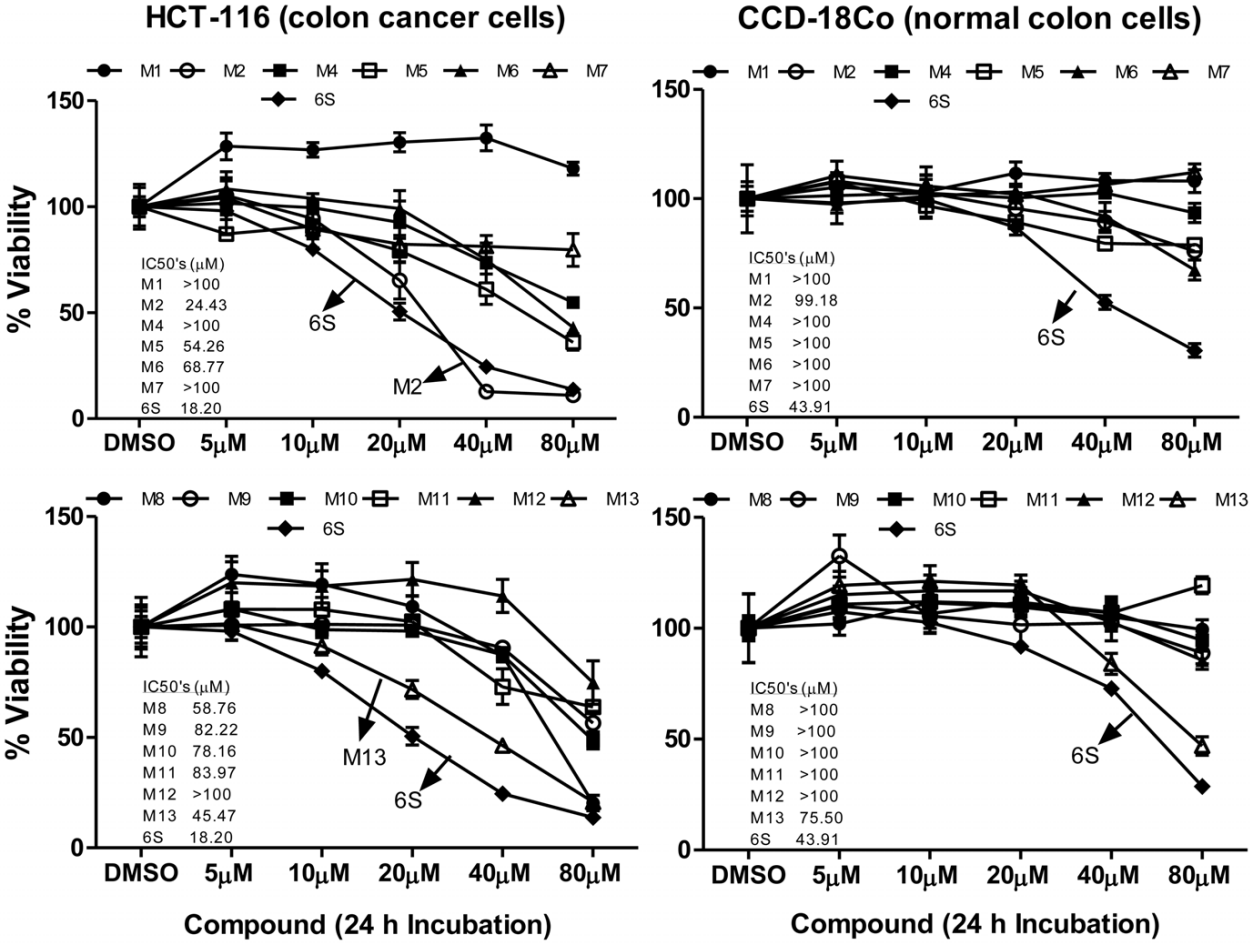
Growth inhibitory effects of [**6**]-shogaol and its metabolites (M1, M2, and M4–M13) against human colon cancer cells HCT-116 and human normal colon cells CCD-18Co. *Bar*, standard error (n = 6).

**Figure 6:**
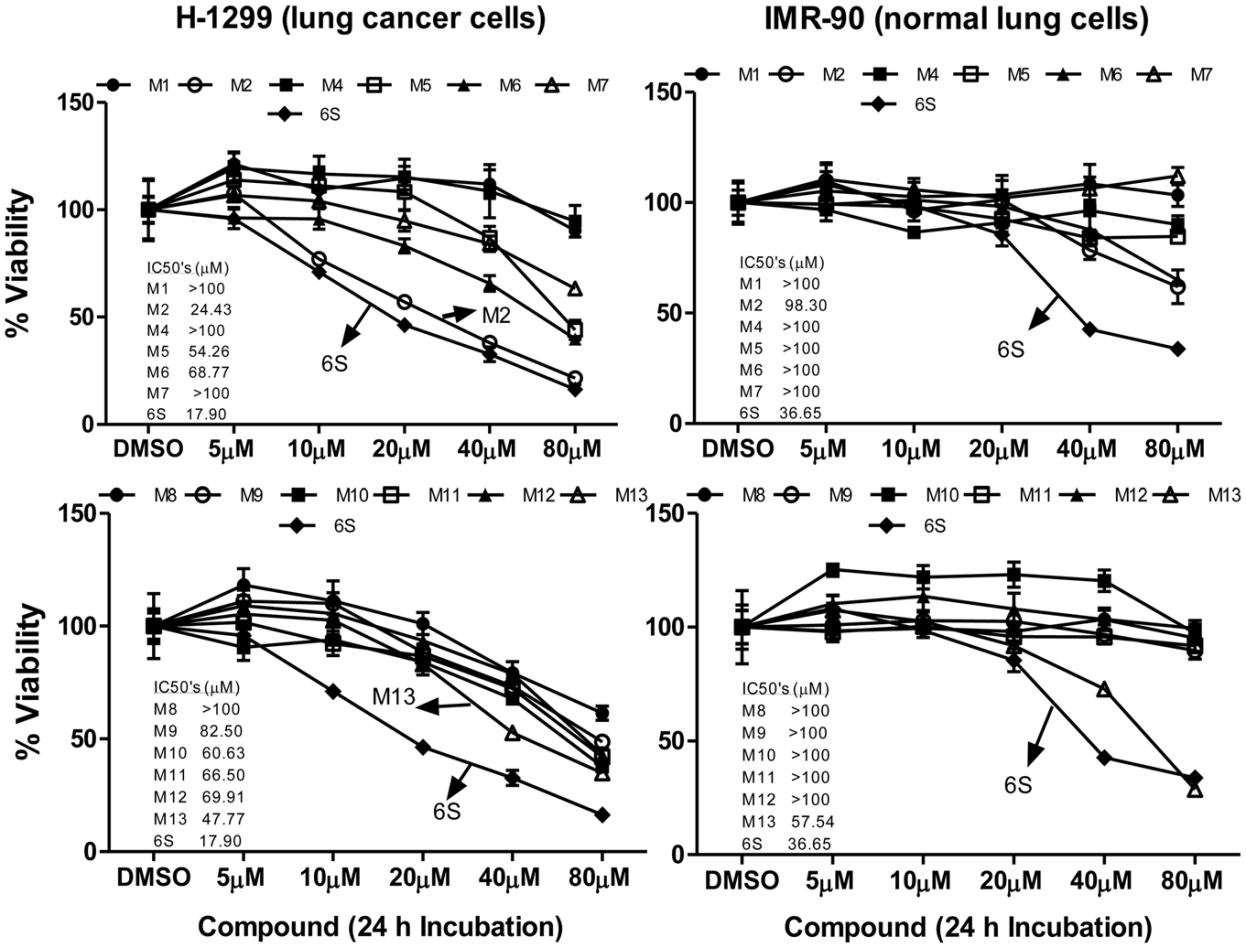
Growth inhibitory effects of [**6**]-shogaol and its metabolites (M1, M2, and M4–M13) against human colon cancer cells H-1299 and human normal lung cells IMR-90. *Bar*, standard error (n = 6).

Two human cancer cell lines, HCT-116 and H-1299, were treated with [6]-shogaol or its synthetic metabolites M1, M2, and M4–M13, with concentrations ranging from 0 to 80 µM. Cell viability assays utilizing MTT resulted in eight active metabolites, M2, M5, M6, M8–M11, and M13, against colon cancer cells HCT-116, with IC_50_ values of 24.43, 54.26, 68.77, 58.76, 82.22, 78.16, 83.97, and 45.47 µM, respectively (Figure 5), and eight active metabolites, M2, M5, M6, and M9–M13, in lung cancer cells H-1299, with IC_50_ values of 25.82, 72.62, 61.28, 82.50, 60.63, 66.50, 69.91, and 47.77 µM, respectively (Figure 6). Among them, M2, the cysteine conjugated metabolite of [6]-shogaol, was found to be most potent toward both HCT-116 and H-1299 cells with IC_50_ values of 24.43 and 25.82 µM, respectively, which was comparable to the parent [6]-shogaol, with an IC_50_ of 18.20 µM in HCT-116 cells and an IC_50_ of 17.90 µM in H-1299 cells. The second most active metabolite was 5-glutathionyl-[6]-shogaol (M13), with IC_50_ values of 45.47 and 47.77 µM in HCT-116 and H-1299 cells, respectively. 5-*N*-acetylcysteinyl-[6]-shogaol (M5) also exhibited noticeable bioactivity with IC_50_ values of 54.26 µM in HCT-116 cells and 72.62 µM in H-1299 cells. This metabolite, however, displayed decreased activity when compared to that of 5-cysteinyl-[6]-shogaol (M2), suggesting the acetylation on α-NH_2_ of the cysteinyl moiety diminishes the activity of M2. Moreover, the reduction of a ketone group on the alkyl side chain resulted in little to no activity against cancer cells HCT-116 and H-1299, as observed from M1 and M4 versus M2 and M5, as well as M6, M9, and M11 versus [6]-shogaol, indicating the reductive biotransformation of [6]-shogaol and its metabolites was primarily inactivating.

Evaluation of cytotoxicity in human normal fibroblast colon cells CCD-18Co and human normal lung cells IMR-90 showed that all synthetic metabolites (M1, M2, and M4–M13) were less toxic than parent [6]-shogaol, and most of them had little to no inhibitory effect (Figures 5 and 6), thereby indicating a detoxifying metabolic biotransformation of [6]-shogaol. More importantly, the metabolite M2, having the greatest potency against both HCT-116 and H-1299 cancer cells, showed almost no toxicity towards normal colon cells CCD-18Co and normal lung cells IMR-90 with IC_50_ values of 99.18 and 98.30 µM, respectively, compared to those of parent [6]-shogaol with an IC_50_ of 43.91 µM toward normal colon cell line CCD-18Co and an IC_50_ of 36.65 µM toward normal lung cell line IMR-90. Moreover, M13, with IC_50_ values of 75.50 and 57.54 µM against cells CCD-18Co and IMR-90, respectively, also displayed lower toxicity compared to parent [6]-shogaol.

**Figure 7:**
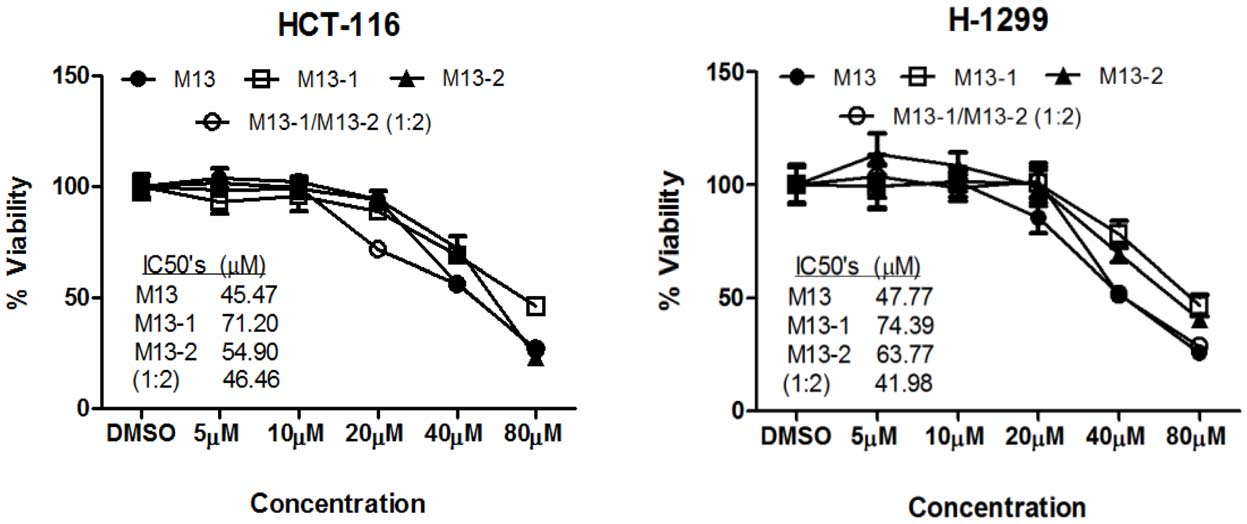
Growth inhibitory effects of [**6**]-shogaol, M13, M13-1, M13-2, and a physical mixture of M13-1 and M13-2 (molar/molar = 1∶2) against human colon cancer cells HCT-116. *Bar*, standard error (n = 6).

In order to investigate the influence of stereochemistry on activity, metabolite M13, as a mixture of diastereomers, was separated by reverse phase prep-HPLC into two individual isomers, M13-1 and M13-2. Cancer cells HCT-116 and H-1299 were treated with M13 or its constituent stereoisomers (M13-1 and M13-2) individually, with concentrations ranging from 0 to 80 µM. We found both isomers had similar but slightly less activity than M13, and M13-2 to be slightly more effective than M13-1, with an IC_50_ value of 54.90 µM in HCT-116 cells and 63.77 µM in H-1299 cells, versus 71.20 µM and 74.39 µM in the same respective cell lines (Figure 7). Reconstituted M13 by individuals M13-1 and M13-2 with an approximate ratio of 1∶2 (molar/molar), which is similar to the ratio in original M13, displayed the equivalent activity, with IC_50_ values of 46.46 µM in HCT-116 cell and 41.98 µM in H-1299 cell, compared to the original M13 with IC_50_ values of 45.47 µM in HCT-116 cells and 47.77 µM in H-1299 cells. This suggested that the observed growth inhibitory effect of M13 could not be attributed to one isomer or the other.

### M2 and M13 Induce Apoptosis in Human Cancer Cells

**Figure 8:**
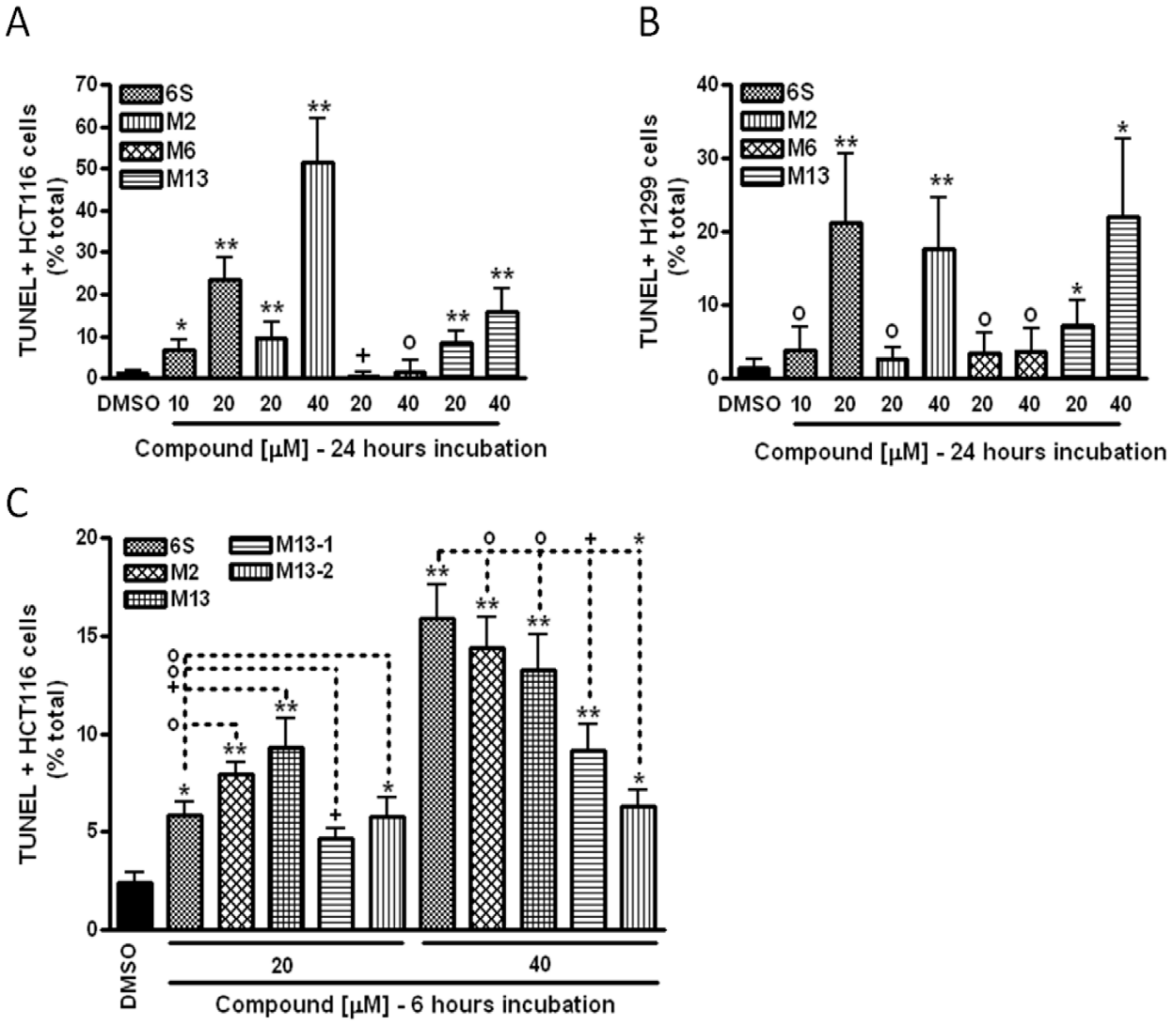
Effects of [**6**]-shogaol and its metabolites M2, M6, and M13 on the induction of apoptosis in HCT-116 (A) and H-1299 (B) cells after 24 hours of incubation; and effects of [**6**]-shogaol, M2, M13, M13-1 and M13-2 on the induction of apoptosis in HCT-116 after 6 hours of incubation (C). TUNEL positive cells have been observed at 400X power. 10 fields per slide have been counted and averaged. *Bar*, standard error; o, not significant; +, p<0.05; *, p<0.01; **: P<0.0001. All statistical tests are unpaired Student t-test, 2 tailed, compared to DMSO or the corresponding [6]-shogaol concentration.

In many cases, cell death is the result of the activation of the major regulatory pathway called apoptosis, or programmed cell death. We aimed to determine whether apoptosis was triggered following exposure to [6]-shogaol and its metabolites by using a TUNEL assay, which detects the DNA breaks characteristic of cells undergoing apoptosis. In this study, we tested the two most active metabolites against cancer cell growth, M2 and M13, as well as one of the most abundant metabolites, M6, together with [6]-shogaol. We incubated H-1299 and HCT-116 cells with them at various concentrations to determine the active range of these compounds versus DMSO only. The results are summarized in Figure 8. After 24 hours of incubation, metabolite M6 did not display any apoptotic effect in HCT-116 and H-1299 cell lines. Significant apoptosis was observed for M2 and M13 in both cell lines, except for M2 in H-1299 cells for the 20 µM concentration. The induction of apoptosis by [6]-shogaol was significantly superior to that of both M2 and M13 at the same concentration (20 µM). In H-1299 cells, an equivalent apoptotic effect to [6]-shogaol could be obtained if the concentration of M2 and M13 was twice (40 µM) than that of [6]-shogaol. Similar results were obtained in HCT-116 cells, except that the M2 apoptotic induction was significantly higher at 40 µM. In all cell lines, an increase in the concentration of [6]-shogaol, M2, M13, M13-1, M13-2 or M6 yielded a corresponding increase in apoptotic level in cancer cells (Figure 8A and 8B).

In order to determine if the apoptotic effect observed in Figure 8A and 8B was constant over time, we incubated [6]-shogaol, M2 and M13 in HCT-116 cells for only 6 hours (Figure 8C). After 6 hours of incubation with [6]-shogaol or metabolites M2 or M13, we were able to detect significantly higher levels of apoptosis for all 3 compounds compared to DMSO in HCT-116 cells. Interestingly there was no significant difference between the apoptotic effect of [6]-shogaol and the effects of M2 at 20 and 40 µM and M13 at 40 µM. M13 was significantly more potent than [6]-shogaol at 20 µM. Exposure of HCT-116 cells to M13 isomers M13-1 and M13-2 also showed a higher level of apoptosis, but the isomers’ apoptotic effect was significantly inferior compared to [6]-shogaol for both concentrations used. These results show that apoptosis is triggered by [6]-shogaol metabolites M2, M13, M13-1 and M13-2, and is the mechanism responsible, at least partially, for the cell death observed previously.

## Discussion

It is now well accepted that natural compounds provide the opportunity to interfere in early stages of cancer or prevent its development altogether [16]. However, these compounds do have their limitations, such as fast *in vivo* turnover, limited quantities in foodstuffs, and their eventual toxicity at higher doses [17]. It is remarkable that anti-cancer effects for these compounds can be observed in cohort studies despite a short half-life and fast metabolism once ingested [18]. Often, presence of the compound can only be assessed by quantification of metabolites, suggesting that these metabolites circulate in the body for a certain amount of time and potentially interact with biological processes [19]. Consequently, one hypothesis explaining the bioactivity of natural compounds is that the metabolites themselves retain and carry part or all of the original compound’s bioactivity. The present study explored this possibility using [6]-shogaol, the main component of dried ginger, and its metabolites.

Our previous study has indicated that [6]-shogaol is extensively metabolized in mice and in cancer cells and more than 90% of [6]-shogaol is converted to its metabolites [12]. We also noticed that human normal cell IMR-90 to some extent metabolized [6]-shogaol in a similar way to cancer cells (data not shown). As a result, fast metabolism of [6]-shogaol might offer the chance for its stable metabolites to get involved in biological processes if they possessed bioactivities. In order to verify this hypothesis, it is critical to obtain the metabolites in a stable form. Isolation from their native environment would not be convenient and most likely would not yield the quantities needed for experimental biology. A chemical synthesis is much more desirable, reproducible, and efficient. We have purified M6–M12 in very limited quantities (0.5–17 mg) from fecal samples collected from [6]-shogaol treated mice [12]. In order to have enough quantity to further study the bioactivity of [6]-shogaol metabolites, we describe the chemistry steps allowing the synthesis of the major metabolites of [6]-shogaol in this study. Twelve metabolites of [6]-shogaol (M1, M2, and M4–M13) were synthesized successfully by practicable approaches. The structures of synthetic metabolites were verified using 1-D and/or 2-D NMR data as well as mass spectra.

We compared the growth inhibitory effects of the synthetic metabolites with [6]-shogaol in two human cancer cells and two human normal cells. Our results showed that two metabolites, M2 and M13, possessed the most comparable growth inhibitory effects to [6]-shogaol towards cancer cells. Most importantly, M2 exhibited a discriminatory effect, as it did not seem to be toxic towards normal cells. This effect was not detected with [6]-shogaol. M13 also showed less toxic effects towards normal cells compared to [6]-shogaol. In addition, M5, M6 and M8–M12 also had certain potency against the growth of cancer cells, but showed no toxicity towards normal cells with IC_50_ values greater than 100 µM (Figures 5 and 6). Our results clearly indicate that metabolites of [6]-shogaol remain bioactive against cancer cells but are much less toxic than [6]-shogaol to normal cells. This confirms our hypothesis that the metabolites themselves retain and carry part or all of the original compound’s bioactivity.

Apoptosis is a mechanism often responsible for the induction of cell death in response to internal or external stress. In order to gain insight into the mechanism of action of the metabolites, we performed a TUNEL assay in HCT-116 cells using M2, M6, M13 and its two isomers, M13-1 and M13-2 and compared their activities to that of [6]-shogaol. It showed that both M2 and M13, but not M6, are capable to induce cancer cell apoptosis in both HCT-116 human colon cancer cells and H-1299 human lung cancer cells (Figure 8A and 8B). For M13, apoptosis induction could not firmly be attributed to one isomer or the other, suggesting that stereo configuration is not important to the bioactivity of this compound. We observed that both M2 and M13 could trigger apoposis in HCT-116 cells at a level similar to that of [6]-shogaol at the 6 hour time point (Figure 8C). However, after 24 hours of exposure to the metabolites, the percentage of TUNEL-positive cells was mostly unchanged at 20 µM for both M2 and M13 while the effect of [6]-shogaol was remarkably increased. The induction effect of M2 on apoptosis at a concentration of 40 µM dramatically increased at the 24 hour time point compared to that of the 6 hour time point, which was significantly higher than that of M13 at a 40 µM concentration. We also observed a concentration-dependant effect of [6]-shogaol and its metabolites on cancer cell apoptosis, where an increase in concentration of a compound resulted in a corresponding increased percentage of apoptotic cells. Altogether these results suggests that it is likely that the activation of others mechanisms are involved in triggering cancer cell death, however, apoptosis appears to be one of the major activated pathways for [6]-shogaol metabolites to induce cancer cell death.

It is always a challenge to separate stereo isomers. Among all the synthesized metabolites, M13 was the only diastereomers mixture that we were able to separate using non-chiral preparative HPLC column. Our results indicated that M13 as the mixture of M13-1 and M13-2 had slightly better growth inhibitory effects on cancer cells than the two diastereomers alone and M13-1 and M13-2 had similar activity, though M13-2 was slightly more potent than M13-1. More importantly, we did observe M13 as the metabolite of [6]-shogaol in the form of a mixture of M13-1 and M13-2 in HCT-116 human colon cancer cells (data not shown). It would be worthwhile to separate the two diastereomers of M2, the most active metabolite of [6]-shogaol, using a chiral column and further determine the effect of stereo configuration on the activity of this compound in future study. We recently identified M2 as a metabolite of [6]-shogoal in humans (unpublished data). Thus, it is important to determine if M2 exists as a mixture of two diastereomers in mice and in humans.

In conclusion, the present study allowed us to show that metabolites of [6]-shogoal can account for the bioactivity of the parent compound, and specifically triggers molecular pathways responsible for cancer cell death in a similar fashion. It suggests that anti-cancer activity attributed to compounds such as [6]-shogoal might have been partly misrepresented in the past. What was originally thought to be an effect of natural compound might have been the direct result of rapid metabolism of the compound, rendering it less capable of triggering molecular mechanisms. However, the apparition of its metabolites in target tissues would then supplement, sustain or replace altogether the bioactive effect of the original compound. Supplementary *in vivo* studies will be needed to obtain a definitive answer to that question, but as of now the study presented here opens novel research possibilities for the study of bioactive metabolites of [6]-shogaol.

## Materials and Methods

### Materials

[6]-Shogaol was purified from ginger extract in our laboratory [8]. All other chemicals were purchased from Sigma (St. Louis, MO) or Thermo Fisher Scientific (Waltham, MA). Anhydrous reactions were carried out in oven-dried glassware under a nitrogen atmosphere unless otherwise noted. Analytical (250 *µ*m thickness, 2–25 *µ*m particle size) and preparative TLC plates (2000 *µ*m thickness, 2–25 *µ*m particle size) were purchased from Sigma (St. Louis, MO) and Sorbent Technologies (Atlanta, GA), respectively. Sephadex LH-20 was purchased from Sigma (St. Louis, MO). HPLC-grade solvents were obtained from VWR Scientific (South Plainfield, NJ). HCT-116 human colon cancer cells, H-1299 human lung cancer cells, CCD-18Co human fibroblast cells derived from colon, IMR-90 human diploid fibroblast cells derived from lung, and Eagle’s minimum essential media (EMEM) were obtained from American Type Tissue Culture (Manassas, VA). McCoy’s 5A medium was purchased from Thermo Fisher Scientific (Waltham, MA). Fetal bovine serum (FBS) and penicillin/streptomycin were purchased from Gemini Bio-Products (West Sacramento, CA). MTT (3-(4,5-dimethylthiaxol-2-yl)-2,5-diphenyltetrazolium bromide) was procured from Calbiochem-Novabiochem (San Diego, CA). Proteinase K was obtained from Ambion (Austin, TX). Apoptag Plus Peroxydase *In Situ* Apoptosis Detection Kit was purchased from Millipore (Billerica, MA).

### Nuclear Magnetic Resonance (NMR)

^1^H, ^13^C NMR, and two-dimensional (2-D) NMR spectra were recorded on a Bruker AVANCE 600 MHz or 700 MHz spectrometer (Brucker, Inc., Silberstreifen, Rheinstetten, Germany). Compounds were analyzed in CDCl_3_ or CD_3_OD. Multiplicities are indicated by s (singlet), d (doublet), t (triplet), q (quartet), and br (broad). The ^13^C NMR spectra are proton decoupled.

### General Procedure A for Michael Addition Reaction

A catalyst amount of NaHCO_3_ (0.05 eq) was added to a mixture of [6]-shogaol (1.0 eq) and amino acid (3.0 eq) in methanol/water (1∶1, v/v). The mixture was stirred at room temperature (rt) for 3–48 h, adjusted pH until 6 with a diluted HOAc solution (0.1 M), and extracted with n-butanol (BuOH) (5 mL × 3). Combined organic layers were concentrated under reduced pressure at 20°C. The residue was subjected to column chromatography (CC) on Sephadex LH-20, and eluted with 90% ethanol in water, producing the desired thiol conjugates M2, M5, or M13.

### General Procedure B for the Synthesis of Ketone Reduced Metabolites using NaBH_4_

NaBH_4_ (2.5–4.0 eq) was added to a solution of M2, M5 or [6]-shogaol (1.0 eq) in methanol at 0°C. After stirring at 0°C for 2 h, the reaction media was neutralized with a diluted HOAc solution (0.1 M) and extracted with n-BuOH (5 mL×3). Combined organic layers were concentrated under reduced pressure. The residue was purified by CC on Sephadex LH-20 or preparative TLC to produce the required compounds M1, M4, or M9.

### Synthesis of 5-cysteinyl-[6]-shogaol (M2)

General procedure A was followed using [6]-shogaol (200 mg, 0.72 mmol), L-cysteine (263 mg, 2.17 mmol), and NaHCO_3_ (3 mg, 0.036 mmol) in methanol/water (10 mL, 1∶1, v/v). The mixture was stirred at rt for 24 h. The final residue was purified by CC on Sephadex LH-20 with 90% ethanol in water to give the title compound M2 as a white solid (170 mg, yield 60%). M2 (a mixture of diastereomers): ^1^H NMR (600 MHz, CD_3_OD) δ 6.77 (1H, d, *J* = 1.5 Hz, H-2'), 6.67 (1H, d, *J* = 8.0 Hz, H-5'), 6.61 (1H, dd, *J* = 8.0, 1.5 Hz, H-6'), 2.77 (2H, m, H-1), 2.74 (2H, m, H-2), 2.71 (1H, m, H-4a), 2.63 (1H, m, H-4b), 3.12 (1H, m, H_minor_-5) and 3.08 (1H, m, H_major_-5), 1.53 (2H, m, H-6), 1.39 (2H, m, H-7), 1.28 (2H, m, H-8), 1.33 (2H, m, H-9), 0.89 (3H, t, *J* = 7.0 Hz, H-10), 3.82 (3H, s, OMe-3'), 3.62 (1H, dd, *J* = 9.3, 3.7 Hz, H*_Cys_*-α, major) and 3.59 (1H, dd, *J* = 9.3, 3.7 Hz, H*_Cys_*-α, minor), 3.18 (1H, dd, *J* = 14.5, 3.7 Hz, H*_Cys_*-βa), and 2.84 (1H, dd, *J* = 14.5, 9.3 Hz, H*_Cys_*-βb); ^13^C NMR (150 MHz, CD_3_OD) δ 133.8 (s, C-1'), 113.1 (d, C-2'), 148.9 (s, C-3'), 145.8 (s, C-4'), 116.2 (d, C-5'), 121.7 (d, C-6'), 30.3 (t, C-1), 47.5 (t, C-2), 211.8 (s, C = O, C-3), 49.6 (t, C-4), 42.3 (2d, C-5), 36.8 (2t, C-6), 27.4 (2t, C-7), 32.6 (2t, C-8), 23.6 (t, C-9), 14.4 (2q, C-10), 56.4 (q, OMe-3'), 56.3 (d, C*_Cys_*-α), 32.8 (2t, C*_Cys_*-β), and 172.5 (s, Cys α-COOH); positive APCIMS: *m/z* 398 [M+H]^+^_._

### Synthesis of 5-cysteinyl-M6 (M1)

General procedure B was followed using M2 (74 mg, 0.19 mmol) and NaBH_4_ (28 mg, 0.75 mmol) in methanol (3 mL). The resulting solution was extracted with n-BuOH (5 mL × 3). Combined organic layers were evaporated under reduced pressure at 20°C. The final residue was purified by CC on Sephadex LH-20 with 90% ethanol in water to give the title compound M1 as a white solid (68 mg, yield 90%); M1 (a mixture of diastereomers): ^1^H NMR (600 MHz, CD_3_OD) δ 6.77 (1H, d, *J* = 1.7 Hz, H-2'), 6.69 (1H, d, *J* = 8.0 Hz, H-5'), 6.62 (1H, dd, *J* = 8.0, 1.7 Hz, H-6'), 2.68 (1H, m, H-1a), 2.58 (1H, m, H-1b), 1.72 (2H, m, H-2), 3.90 (1H, m, H_minor_-3) and 3.66 (1H, m, H_major_-3), 1.71 (2H, m, H-4), 2.94 (1H, m, H-5), 1.66 (1H, m, H-6a), 1.52 (1H, m, H-6b), 1.44 (2H, m, H-7), 1.28 (2H, m, H-8), 1.33 (2H, m, H-9), 0.89 (3H, t, *J* = 7.0 Hz, H-10), 3.82 (3H, s, OMe-3'), 3.64 (1H, m, H*_Cys_*-α), 3.15 (1H, m, H*_Cys_*-βa), and 2.85 (1H, m, H*_Cys_*-βb); ^13^C NMR (150 MHz, CD_3_OD) δ 135.1 (s, C-1'), 113.2 (d, C-2'), 148.8 (s, C-3'), 145.5 (s, C-4'), 116.1 (d, C-5'), 121.8 (d, C-6'), 32.5 (2t, C-1), 41.1 (2t, C-2), 69.3 (2d, C-3), 43.9 (t, C-4), 43.8 (2d, C-5), 35.0 (2t, C-6), 27.1 (2t, C-7), 33.0 (t, C-8), 23.6 (t, C-9), 14.4 (q, C-10), 56.4 (q, OMe-3'), 55.8 (2d, C*_Cys_*-α), 32.8 (4t, C*_Cys_*-β), and 172.8 (s, Cys α-COOH); positive APCIMS: *m/z* 400 [M+H]^+^_._

### Synthesis of 5-*N*-acetylcysteinyl-[6]-shogaol (M5)

General procedure A was followed using [6]-shogaol (200 mg, 0.72 mmol), N-acetyl-*L*-cysteine (354 mg, 2.17 mmol), and NaHCO_3_ (3 mg, 0.036 mmol) in methanol/water (10 mL, 1∶1, v/v). The mixture was stirred at rt for 72 h. The final residue was purified by CC on Sephadex LH-20 with 90% ethanol in water to give title compound M5 as a white solid (252 mg, yield 80%); M5 (a mixture of diastereomers): ^1^H NMR (600 MHz, CD_3_OD) 6.77 (1H, d, *J* = 1.7 Hz, H-2'), δ 6.67 (1H, d, *J* = 8.0 Hz, H-5'), 6.61 (1H, dd, *J* = 8.0, 1.7 Hz, H-6'), 2.78 (2H, m, H-1), 2.77 (2H, m, H-2), 2.70 (1H, dd, *J* = 16.8, 8.1 Hz, H-4a), 2.64 (1H, dd, *J* = 16.8, 6.3 Hz, H-4b), 3.10 (1H, m, H-5), 1.48 (2H, m, H-6), 1.38 (2H, m, H-7), 1.25 (2H, m, H-8), 1.28 (2H, m, H-9), 0.89 (3H, t, *J* = 7.0 Hz, H-10), 3.82 (3H, s, OMe-3'), 4.58 (1H, dd, *J* = 8.1, 4.8 Hz, H*_Cys_*-α, major) and 4.53 (1H, dd, *J* = 8.1, 4.8 Hz, H*_Cys_*-α, minor), 3.02 (1H, dd, *J* = 13.6, 4.8 Hz, H*_Cys_*-βa, minor) and 2.96 (1H, dd, *J* = 13.6, 4.8 Hz, H*_Cys_*-βa, major), 2.89 (1H, dd, *J* = 13.6, 7.2 Hz, H*_Cys_*-βb, major) and 2.76 (1H, dd, *J* = 13.6, 7.2 Hz, H*_Cys_*-βb, minor), and 2.01 (3H, s, CH_3_CO, major) and 1.98 (3H, s, CH_3_CO, minor); positive APCIMS: *m/z* 440 [M+H]^+^_._

### Synthesis of 5-*N*-acetylcysteinyl-M6 (M4)

General procedure B was followed using **M5** (151 mg, 0.34 mmol) and NaBH_4_ (53 mg, 1.38 mmol) in methanol (10 mL). The resulting solution was extracted with n-BuOH (10 mL × 3). Combined organic layers were evaporated under reduced pressure at 20°C. The final residue was purified by CC on Sephadex LH-20 with 90% ethanol in water to give the title compound M4 as a white solid (100 mg, yield 66%); M4 (a mixture of diastereomers): ^1^H NMR (600 MHz, CD_3_OD) δ 6.77 (1H, brs, H-2'), 6.68 (1H, d, *J* = 8.0 Hz, H-5'), 6.62 (1H, dd, *J* = 8.0 Hz, H-6'), 2.67 (1H, m, H-1a), 2.58 (1H, m, H-1b), 1.72 (2H, m, H-2), 3.88 (1H, m, H_minor_-3) and 3.70 (1H, m, H_major_-3), 1.68 (2H, m, H-4), 2.83 (1H, m, H-5), 1.60 (1H, m, H-6a), 1.44 (1H, m, H-6b), 1.45 (2H, m, H-7), 1.28 (2H, m, H-8), 1.33 (2H, m, H-9), 0.89 (3H, t, *J* = 7.0 Hz, H-10), 3.83 (3H, s, OMe-3'), 4.54 (1H, m, H*_Cys_*-α), 3.00 (1H, m, H*_Cys_*-βa), 2.80 (1H, m, H*_Cys_*-βb), and 1.98 (3H, s, CH_3_CO); ^13^C NMR (150 MHz, CD_3_OD) δ 135.1 (2s, C-1'), 113.2 (d, C-2'), 148.8 (s, C-3'), 145.5 (s, C-4'), 116.1 (d, C-5'), 121.8 (d, C-6'), 32.5 (t, C-1), 41.0 (2t, C-2), 69.3 (4d, C-3), 44.3 (4t, C-4), 43.8 (2d, C-5), 35.2 (t, C-6), 27.2 (4t, C-7), 32.9 (t, C-8), 23.6 (t, C-9), 14.4 (2q, C-10), 56.4 (q, OMe-3'), 54.2 (2d, C*_Cys_*-α), 32.7 (2t, C*_Cys_*-β), 173.2 (s, Cys α-COOH), 174.0 (s, CH_3_ CO), and 22.4 (q, CH_3_CO); positive APCIMS: *m/z* 442 [M+H]^+^_._

### Synthesis of 1-(4'-hydroxy-3'-methoxyphenyl)-4-decen-3-ol (M6)

A solution of [6]-shogaol (138 mg, 0.5 mmol) in methanol (10 mL) was cooled to −78°C, CeCl_3_.7H_2_O (745 mg, 2.0 mmol) was added and the mixture was stirred at −78°C for 10 min. Then, NaBH_4_ (48 mg, 1.25 mmol) was added to the mixture and allowed to react at −78°C for 30 min. The reaction was quenched by saturated aqueous NH_4_Cl solution (20 mL) and extracted with ethyl acetate (20 mL × 3). The organic phases were separated, pooled, washed with water (10 mL × 2) and brine (10 mL × 1), dried over Na_2_SO_4_, and evaporated in vacuo. The residue was subjected to preparative TLC (hexane/EtOAc = 3∶1) to produce the title compound M6 as a colorless oil (139 mg, yield 100%); ^1^H NMR (600 MHz, CDCl_3_) δ 6.70 (1H, d, *J* = 1.7 Hz, H-2'), 6.82 (1H, d, *J* = 8.0 Hz, H-5'), 6.68 (1H, dd, *J* = 8.0, 1.7 Hz, H-6'), 2.62 (2H, m, H-1), 1.85 (1H, m, H-2a), 1.78 (1H, m, H-2b), 4.07 (1H, m, H-3), 5.49 (1H, dd, *J* = 15.3, 6.7 Hz, H-4), 5.65 (1H, dt, *J* = 15.3, 6.7 Hz, H-5), 2.03 (2H, m, H-6), 1.38 (2H, m, H-7), 1.32–1.25 (4H, m, H-8 and H-9), 0.89 (3H, t, *J* = 6.9 Hz, H-10), and 3.87 (3H, s, OMe-3'); positive APCIMS: *m/z* 279 [M+H]^+^_._

### Synthesis of 5-methoxy-1-(4'-hydroxy-3'-methoxyphenyl)-decan-3-one (M7)

A solution of [6]-shogaol (100 mg, 0.36 mmol) in methanol (5 mL) at 0°C was treated with a solution of sodium (21 mg, 0.91 mmol) in methanol (1 mL). After 4.0 h, glacial acetic acid (0.5 mL) was added, and the solution was concentrated under reduced pressure. The residue was dissolved in water (5 mL), and extracted with ethyl acetate (5 mL × 3). The organic phases were pooled, washed with water (5 mL × 2) and brine (5 mL × 1), dried over Na_2_SO_4_, and evaporated in vacuo. The residue was subjected to preparative TLC (hexane/EtOAc = 4∶1) to give the title compound M7 as a yellow oil (100 mg, yield 90%); ^1^H NMR (600 MHz, CDCl_3_) δ 6.69 (1H, d, *J* = 1.6 Hz, H-2'), 6.82 (1H, d, *J* = 8.1 Hz, H-5'), 6.66 (1H, dd, *J* = 8.1, 1.6 Hz, H-6'), 2.75 (2H, m, H-1), 2.83 (2H, t, *J* = 7.5 Hz, H-2), 2.64 (1H, dd, *J* = 15.7, 7.6 Hz, H-4a), 2.40 (1H, dd, *J* = 15.7, 4.7 Hz, H-4b), 3.66 (1H, m, H-5), 1.48 (1H, m, H-6a), 1.42 (1H, m, H-6b), 1.31–1.25 (6H, m, ranged from H-7 to H-9), 0.88 (3H, t, *J* = 7.1 Hz, H-10), and 3.87 (3H, s, OMe-3'); positive APCIMS, *m/z* 309 [M+H]^+^_._

### Synthesis of 5-methylthio-1-(4'-hydroxy-3'-methoxyphenyl)-decan-3-one (M10)

A solution of NaSCH_3_ in water (15% w/w, 1.5 mL, 3.19 mmol) was added to a solution of [6]-shogaol (100 mg, 0.36 mmol) in THF (10 mL) at rt in portions. After stirred for 6.0 h, 10 mL of water was added, followed by extraction with ethyl acetate (10 mL × 3). The organic phases were separated, pooled, washed with water (10 mL × 2) and brine (10 mL × 1), dried over Na_2_SO_4_, and evaporated in vacuo. The residue was loaded to preparative HPLC (methanol in water: 70%–100% in 50 min) to give the title compound M10 as a yellow oil (70 mg, yield 60%); ^1^H NMR (600 MHz, CDCl_3_) δ 6.69 (1H, d, *J* = 1.6 Hz, H-2'), 6.82 (1H, d, *J* = 8.0 Hz, H-5'), 6.67 (1H, dd, *J* = 8.0, 1.6 Hz, H-6'), 2.73 (2H, m, H-1), 2.84 (2H, t, *J* = 7.6 Hz, H-2), 2.67 (1H, dd, *J* = 16.6, 7.5 Hz, H-4a), 2.57 (1H, dd, *J* = 16.6, 6.4 Hz, H-4b), 3.02 (1H, m, H-5), 1.49 (2H, m, H-6), 1.42 (1H, m, H-7a), 1.36 (1H, m, H-7b), 1.32–1.24 (4H, m, H-8 and H-9), 0.88 (3H, t, *J* = 6.9 Hz, H-10), 3.87 (3H, s, OMe-3'), and 2.03 (3H, s, SCH_3_-5); positive APCIMS: *m/z* 325 [M+H]^+^_._

### Synthesis of 1-(4'-hydroxy-3'-methoxyphenyl)-decan-3-one (M11)

A solution of [6]-shogaol (276 mg, 1.0 mmol) in THF (2 mL) at rt was treated with 10% Pd/C (30 mg, 10% w/w) under H_2_. The mixture was stirred at rt overnight and filtered. The filtrate was concentrated under reduced pressure. The residue was loaded to preparative TLC (hexane/EtOAc = 8∶1) to give the title compound M11 as a yellow oil (272 mg, yield 98%); ^1^H NMR (600 MHz, CDCl_3_) δ 6.69 (1H, d, *J* = 1.6 Hz, H-2'), 6.82 (1H, d, *J* = 8.0 Hz, H-5'), 6.66 (1H, dd, *J* = 8.0, 1.7 Hz, H-6'), 2.69 (2H, t, *J* = 7.4 Hz, H-1), 2.82 (2H, t, *J* = 7.4 Hz, H-2), 2.37 (1H, t, *J* = 7.4 Hz, H-4), 1.54 (2H, m, H-5), 1.30–1.24 (8H, m, ranged from H-6 to H-9), 0.87 (3H, t, *J* = 6.8 Hz, H-10), and 3.87 (3H, s, OMe-3'); positive APCIMS, *m/z* 279 [M+H]^+^_._

### Synthesis of 1-(3',4'-dihydroxyphenyl)-decan-3-one (M8)

A solution of BBr_3_ in DCM (1.0 M, 0.67 mL, 0.67 mmol) was added dropwise to a solution of M11 (74 mg, 0.27 mmol) in dichloromethane (DCM) (3 mL) at −78°C. The reaction was allowed to warm up to rt for 2.0 h, quenched with water (10 mL), and extracted with ethyl acetate (10 mL × 3). The organic phases were separated, pooled, washed with water (10 mL × 2) and brine (10 mL × 1), dried over Na_2_SO_4_, and evaporated in vacuo. The residue was subjected to preparative TLC (DCM/Methanol = 20∶1) to give the title compound M8 as a red solid (50 mg, yield 70%); ^1^H NMR (600 MHz, CDCl_3_) δ 6.70 (1H, d, *J* = 1.9 Hz, H-2'), 6.76 (1H, d, *J* = 8.0 Hz, H-5'), 6.59 (1H, dd, *J* = 8.0, 1.9 Hz, H-6'), 2.69 (2H, t, *J* = 7.4 Hz, H-1), 2.78 (2H, t, *J* = 7.4 Hz, H-2), 2.37 (2H, t, *J* = 7.4 Hz, H-4), 1.54 (2H, m, H-5), 1.30–1.24 (8H, m, ranged from H-6 to H-9), and 0.87 (3H, t, *J* = 6.8 Hz, H-10); positive APCIMS: *m/z* 265 [M+H]^+^_._

### Synthesis of 1-(4'-hydroxy-3'-methoxyphenyl)-decan-3-ol (M9)

General procedure B was followed using **M11** (100 mg, 0.36 mmol) and NaBH_4_ (34 mg, 0.90 mmol) in methanol (2 mL). The resulting solution was extracted with ethyl acetate (5 mL × 3). Combined organic layers were concentrated under reduced pressure. The final residue was purified by preparative TLC (DCM/Methanol = 40∶1) to produce the title compound M9 as a white solid (90 mg, yield 90%); ^1^H NMR (600 MHz, CDCl_3_) δ 6.71 (1H, d, *J* = 1.6 Hz, H-2'), 6.83 (1H, d, *J* = 8.0 Hz, H-5'), 6.69 (1H, dd, *J* = 8.0, 1.6 Hz, H-6'), 2.72 (1H, m, H-1a), 2.60 (1H, m, H-1b), 1.76 (1H, m, H-2a), 1.71 (1H, m, H-2b), 3.62 (1H, m, H-3), 1.48 (2H, m, H-4), 1.44 (2H, m, H-5), 1.32–1.26 (8H, m, ranged from H-6 to H-9), 0.88 (3H, t, *J* = 6.8 Hz, H-10), and 3.88 (3H, s, OMe-3'); positive APCIMS: *m/z* 281 [M+H]^+^_._

### Synthesis of 5-methylthio-1-(4'-Hydroxy-3'-methoxyphenyl)-decan-3-ol (M12)

General procedure B was followed using M10 (39 mg, 0.12 mmol) and NaBH_4_ (11 mg, 0.30 mmol) in methanol (3 mL). The resulting solution was extracted with ethyl acetate (5 mL × 3). The combined organic layers were concentrated under reduced pressure. The final residue was purified by preparative TLC (DCM/Methanol = 50∶1) to produce the title compound M12 as a yellow oil (39 mg, yield 100%); Mixture of diastereomers: ^1^H NMR (600 MHz, CDCl_3_) δ 6.71 (1H, d, *J* = 1.5 Hz, H-2'), 6.82 (1H, d, *J* = 8.0 Hz, H-5'), 6.68 (1H, dd, *J* = 8.0, 1.5 Hz, H-6'), 2.74 (1H, m, H-1a), 2.61 (1H, m, H-1b), 1.75 (2H, m, H-2), 3.98 (1H, m, H_minor_-3) and 3.80 (1H, m, H_maior_-3), 1.70 (1H, m, H-4a), 1.65 (1H, m, H-4b), 2.75 (1H, m, H-5), 1.61 (2H, m, H-6), 1.44 (2H, m, H-7), 1.32–1.23 (4H, m, H-8 and H-9), 0.88 (3H, t, *J* = 7.0 Hz, H-10), 3.86 (3H, s, OMe-3'), and 2.02 (3H, s, SMe-5); positive APCIMS, *m/z* 327 [M+H]^+^_._

### Synthesis of 5-glutathiol-[6]-shogaol (M13)

General procedure A was followed using [6]-shogaol (100 mg, 0.36 mmol), reduced *L*-glutathione (333 mg, 1.09 mmol), and NaHCO_3_ (1.5 mg, 0.018 mmol) in methanol/water (8 mL, 1∶1, v/v). The mixture was stirred at rt for 3 h. The final residue was purified by CC on Sephadex with 90% ethanol in water to give the title compound M13 as a white solid (168 mg, yield 80%); M13 (a mixture of diastereomers): ^1^H NMR (600 MHz, CD_3_OD) δ 6.77 (1H, d, *J* = 1.6 Hz, H-2'), 6.68 (1H, d, *J* = 8.0 Hz, H-5'), 6.61 (1H, dd, *J* = 8.0, 1.6 Hz, H-6'), 2.78–2.75 (4H, m, H-1 and H-2), 2.74–2.61 (2H,m, H-4), 3.10 (1H, m, H-5), 1.51–1.45 (2H, m, H-6), 1.42–1.33 (2H, m, H-7), 1.25 (2H, m, H-8), 1.28 (2H, m, H-9), 0.88 (3H, t, *J* = 7.3 Hz, H-10), 3.82 (3H, s, OMe-3'), 3.65 (1H, m, H*_Glu_*-α), 2.13 (2H, m, H*_Glu_*-β), 2.55 (1H, m, H*_Glu_*-γa), 2.51 (1H, m, H*_Glu_*-γb), 4.50 (1H, dd, *J* = 8.5, 5.1 Hz, H*_Cys_*-α), 3.05–2.95 (1H, m, H*_Cys_*-βa), 2.84–2.80 (1H, m, H*_Cys_*-βb), and 3.80 (2H, brs, H*_Gly_*-α); ^13^C NMR (150 MHz, CD_3_OD) δ 133.9 (s, C-1'), 113.2 (d, C-2'), 148.9 (s, C-3'), 145.7 (s, C-4'), 116.2 (d, C-5'), 121.8 (d, C-6'), 30.4 (t, C-1), 46.0 (2t, C-2), 211.2 (s, C = O, C-3), 49.8 (2t, C-4), 42.2 (2d, C-5), 36.2 (2t, C-6), 27.5 (2t, C-7), 32.8 (t, C-8), 23.6 (t, C-9), 14.4 (q, C-10), 56.4 (q, OMe-3'), 55.4 (d, C*_Glu_*-α), 27.8 (t, C*_Glu_*-β), 33.0 (t, C*_Glu_*-γ), 174.0 (s, Glu α-COOH), 175.2 (s, Glu γ-CON), 55.0 (2d, C*_Cys_*-α), 33.3 (2t, C*_Cys_*-β), 172.9 (s, Cys α-CON), 45.0 (t, C*_Gly_*-α), and 175.2 (s, Gly α-COOH); positive APCIMS: *m/z* 584 [M+H]^+^_._

### Separation of the M13 Isomers Using Preparative HPLC

Waters preparative HPLC systems with 2545 binary gradient module, Waters 2767 sample manager, Waters 2487 autopurification flow cell, Waters fraction collector III, dual injector module, and 2489 UV/Visible detector, were used to separate M13 isomers. A Phenomenex Gemini-NX C_18_ column (250 mm × 30.0 mm i.d., 5 *µ*m) was used with a flow rate of 20.0 mL/min. The wavelength of UV detector was set at 280 nm. The injection volume was 1.0 mL for each run. The mobile phase consisted of solvent A (H_2_O +0.1% formic acid) and solvent B (MeOH +0.1% formic acid).

M13 (5 mg/mL) was injected to the preparative column and eluted with a gradient solvent system (0% B from 0 to 5 min; 0 to 50% B from 5 to 15 min; 50 to 60% B from 15 to 25 min; 60 to 80% B from 25 to 45 min; then 0% B from 45 to 50 min). The fractions were checked by a HPLC-APCI-MS system and then combined. A total of 7 runs resulted in 10 mg of M13-1 (t*_R_* 16.5 min) and 22 mg of M13-2 (t*_R_* 16.8 min). M13-1**:** white solid; ^1^H NMR (700 MHz, CD_3_OD) δ 6.77 (1H, d, *J* = 1.8 Hz, H-2'), 6.69 (1H, d, *J* = 8.1 Hz, H-5'), 6.61 (1H, dd, *J* = 8.1, 1.8 Hz, H-6'), 2.78 (2H, m, H-1), 2.77 (2H, m, H-2), 2.76 (1H, dd, *J* = 17.1, 7.2 Hz, H-4a), 2.65 (1H, dd, *J* = 17.1, 6.4 Hz, H-4b), 3.10 (1H, m, H-5), 1.49 (2H, m, H-6), 1.42 (1H, m, H-7a), 1.31 (1H, m, H-7b), 1.25 (2H, m, H-8), 1.28 (2H, m, H-9), 0.88 (3H, t, *J* = 7.1 Hz, H-10), 3.82 (3H, s, OMe-3'), 3.62 (1H, t, *J* = 6.0 Hz, H*_Glu_*-α), 2.13 (2H, m, H*_Glu_*-β), 2.55 (1H, m, H*_Glu_*-γa), 2.50 (1H, m, H*_Glu_*-γb), 4.49 (1H, dd, *J* = 8.7, 5.0 Hz, H*_Cys_*-α), 3.00 (1H, dd, *J* = 13.7, 5.0 Hz, H*_Cys_*-βa), 2.73 (1H, dd, *J* = 13.7, 8.7 Hz, H*_Cys_*-βb), and 3.74 (2H, AB, *J* = 17.2 Hz, H*_Gly_*-α); ^13^C NMR (175 MHz, CD_3_OD) δ 134.0 (s, C-1'), 113.2 (d, C-2'), 148.9 (s, C-3'), 145.7 (s, C-4'), 116.2 (d, C-5'), 121.8 (d, C-6'), 30.4 (t, C-1), 46.1 (t, C-2), 211.3 (s, C = O, C-3), 49.9 (t, C-4), 42.0 (d, C-5), 36.2 (t, C-6), 27.6 (t, C-7), 32.8 (t, C-8), 23.6 (t, C-9), 14.4 (q, C-10), 56.4 (q, OMe-3'), 55.6 (d, C*_Glu_*-α), 27.9 (t, C*_Glu_*-β), 33.1 (t, C*_Glu_*-γ), 174.3 (s, Glu α-COOH), 175.2 (s, Glu γ-CON), 55.0 (d, C*_Cys_*-α), 33.2 (t, C*_Cys_*-β), 172.4 (s, Cys α-CON), 44.5 (t, C*_Gly_*-α), and 175.9 (s, Gly α-COOH); positive APCIMS: *m/z* 584 [M+H]^+^_._ M13-2: white solid; ^1^H NMR (700 MHz, CD_3_OD) δ 6.77 (1H, d, *J* = 1.8 Hz, H-2'), 6.69 (1H, d, *J* = 8.1 Hz, H-5'), 6.61 (1H, dd, *J* = 8.1, 1.8 Hz, H-6'), 2.77 (2H, m, H-1), 2.76 (2H, m, H-2), 2.70 (1H, dd, *J* = 17.1, 7.2 Hz, H-4a), 2.64 (1H, dd, *J* = 17.1, 6.4 Hz, H-4b), 3.10 (1H, m, H-5), 1.48 (2H, m, H-6), 1.38 (2H, m, H-7), 1.25 (2H, m, H-8), 1.28 (2H, m, H-9), 0.88 (3H, t, *J* = 7.1 Hz, H-10), 3.82 (3H, s, OMe-3'), 3.64 (1H, t, *J* = 6.0 Hz, H*_Glu_*-α), 2.14 (2H, m, H*_Glu_*-β), 2.56 (1H, m, H*_Glu_*-γa), 2.50 (1H, m, H*_Glu_*-γb), 4.49 (1H, dd, *J* = 8.7, 5.0 Hz, H*_Cys_*-α), 2.97 (1H, dd, *J* = 13.7, 5.0 Hz, H*_Cys_*-βa), 2.81 (1H, dd, *J* = 13.7, 8.7 Hz, H*_Cys_*-βb), and 3.74 (2H, AB, *J* = 17.2 Hz, H*_Gly_*-α); ^13^C NMR (175 MHz, CD_3_OD) δ 133.9 (s, C-1'), 113.2 (d, C-2'), 148.8 (s, C-3'), 145.7 (s, C-4'), 116.2 (d, C-5'), 121.8 (d, C-6'), 30.4 (t, C-1), 46.0 (t, C-2), 211.3 (s, C = O, C-3), 49.6 (t, C-4), 42.4 (d, C-5), 36.3 (t, C-6), 27.5 (t, C-7), 32.8 (t, C-8), 23.6 (t, C-9), 14.4 (q, C-10), 56.4 (q, OMe-3'), 55.5 (d, C*_Glu_*-α), 27.9 (t, C*_Glu_*-β), 33.1 (t, C*_Glu_*-γ), 174.3 (s, Glu α-COOH), 175.3 (s, Glu γ-CON), 55.1 (d, C*_Cys_*-α), 33.3 (t, C*_Cys_*-β), 172.4 (s, Cys α-CON), 44.3 (t, C*_Gly_*-α), and 175.9 (s, Gly α-COOH); positive APCIMS: *m/z* 584 [M+H]^+^_._

### Growth Inhibition of Human Cancer and Normal Cells

Cell growth inhibition was determined by a MTT colorimetric assay [20]. Human colon cancer cells HCT-116, human lung cancer cells H-1299, human colon fibroblast cells CCD-18Co, and human lung fibroblast cells IMR-90 were plated in 96-well microtiter plates with 3000 cells/well and allowed to attach for 24 h at 37°C. The test compounds (in DMSO) were added to cell culture medium to the desired final concentrations (final DMSO concentrations for control and treatments were 0.1%). After the cells were cultured for 24 h, the medium was aspirated and cells were treated with 200 µL fresh medium containing 2.41 mmol/L MTT. After incubation for 3 h at 37°C, the medium containing MTT was aspirated, 100 µL of DMSO was added to solubilize the formazan precipitate, and plates were shaken gently for an hour at room temperature. Absorbance values were derived from the plate reading at 550 nm on a Biotek microtiter plate reader (Winooski, VT). The reading reflected the number of viable cells and was expressed as a percentage of viable cells in the control. Both HCT-116 and H-1299 cells were cultured in McCoy’s 5A medium. CCD-18Co and IMR-90 cells were cultured in Eagle’s modified essential medium (EMEM). All of the above media were supplemented with 10% fetal bovine serum, 1% penicillin/streptomycin, and 1% glutamine, and the cells were kept in a 37°C incubator with 95% humidity and 5% CO_2_.

### TUNEL (Terminal Deoxynucleotidyl Transferase dUTP Nick end Labeling) Assay

HCT-116 and H1299 cells were seeded in 6-well plates at 1.10^5^ cells/well and incubated at 37°C in 5% CO_2_ incubator. After 24 hours, fresh media supplemented with DMSO (control), [6]-shogaol, M2, M6, or M13 metabolites (20 µM or 40 µM) were added to the wells. After 6 or 24 hours incubation at 37°C in 5% CO_2_ incubator, cells were washed and pre-treated for 15 min at room temperature with a solution of 20 µg/ml proteinase K. Cells were then washed twice with phosphate buffer saline pH 7.4 (PBS) and fixed for 10 min at room temperature using 10% neutral formaldehyde solution. After 2 washes in ddH_2_O, cells were resuspended in 100 µL ddH_2_O and applied on silanized microscope slides. Slides were incubated overnight at 37°C, and washed twice with PBS. TUNEL assay was then carried out according to the manufacturer’s protocol. Cells were observed under 400X power using a Zeiss microscope A1 (Thornwood, NY). 10 fields per slide were evaluated, and TUNEL+ cells (with brown coloration in the nucleus) were expressed as a percentage of the total number of cells contained in a field.

### Statistical Analysis

For simple comparisons between two groups, two-tailed Student's *t*-test was used. A p-value of less than 0.05 was considered statistically significant in all the tests.
